# Transforming Growth Factor (TGF) β and Endometrial Vascular Maturation

**DOI:** 10.3389/fcell.2021.640065

**Published:** 2021-04-09

**Authors:** Qinsheng Lu, Dingqian Sun, Sourima Biswas Shivhare, Huomei Hou, Judith N. Bulmer, Barbara A. Innes, Dharani K. Hapangama, Gendie E. Lash

**Affiliations:** ^1^Division of Uterine Vascular Biology, Guangzhou Institute of Pediatrics, Guangzhou Women and Children’s Medical Center, Guangzhou Medical University, Guangzhou, China; ^2^Reproductive and Vascular Biology Group, Institute of Cellular Medicine, Newcastle University, Newcastle upon Tyne, United Kingdom; ^3^Department of Women’s and Children’s Health, Institute of Life Course and Medical Sciences, University of Liverpool, Liverpool Women’s Hospital, Liverpool, United Kingdom

**Keywords:** endometrium, heavy menstrual bleeding, recurrent pregnancy loss, transforming growth factor beta, VSMC differentiation

## Abstract

Appropriate growth and development of the endometrium across the menstrual cycle is key for a woman’s quality of life and reproductive well-being. Recurrent pregnancy loss (RPL) and heavy menstrual bleeding (HMB) affect a significant proportion of the female population worldwide. These endometrial pathologies have a significant impact on a woman’s quality of life as well as placing a high economic burden on a country’s health service. An underlying cause for both conditions is unknown in approximately 50% of cases. Previous research has demonstrated that aberrant endometrial vascular maturation is associated with both RPL and HMB, where it is increased in RPL but reduced in HMB. TGFβ1 is one of the key growth factors that regulate vascular maturation, by inducing phenotypic switching of vascular smooth muscle cells (VSMCs) from a synthetic phenotype to a more contractile one. Our previous data demonstrated an increase in TGFβ1 in the endometrium of RPL, while others have shown a decrease in women with HMB. However, TGFβ1 bioavailability is tightly controlled, and we therefore sought to perform an extensive immunohistochemical analysis of different components in the pathway in the endometrium of normal controls, women with HMB or RPL. In addition, two *in vitro* models were used to examine the role of TGFβ1 in endometrial vascular maturation and endothelial cell (EC):VSMC association. Taken all together, the immunohistochemical data suggest a decrease in bioavailability, receptor binding capacity, and signaling in the endometrium of women with HMB compared with controls. In contrast, there is an increase in the bioavailability of active TGFβ1 in the endometrium of women with RPL compared with controls. Endometrial explants cultured in TGFβ1 had an increase in the number of vessels associated with contractile VSMC markers, although the total number of vessels did not increase. In addition, TGFβ1 increased EC:VSMC association in an *in vitro* model. In conclusion, TGFβ1 is a key regulator of endometrial vascular maturation and could be considered as a therapeutic target for women suffering from HMB and/or RPL.

## Introduction

The endometrium is one of the most dynamic tissues in the human body, undergoing a repetitive cycle of proliferation, differentiation and breakdown every month during the menstrual cycle. After menstruation, the endometrium functionalis is regenerated from the remaining endometrium basalis, a niche for endometrial stem cells and stumps of the endometrial vasculature. Increasing evidence suggests that dysregulated regeneration of the endometrium may contribute to conditions affecting a woman’s reproductive health, e.g., recurrent pregnancy loss (RPL) and heavy menstrual bleeding (HMB). Abnormal development and flow in these endometrial blood vessels has been implicated in various reproductive disorders including RPL, recurrent implantation failure (RIF), unexplained infertility, and HMB (Goswamy et al., 1988; Steer et al., 1994; Habara et al., 2002; Quenby et al., 2009).

Heavy menstrual bleeding (HMB) affects approximately 10 million women annually in the United States, including 30% of women of reproductive age, and significantly impacts a woman’s quality of life and impacts society in terms of health care costs and lost productivity of the female workforce (Hapangama and Bulmer, 2016; NICE, 2018; Critchley et al., 2020). While HMB may result from clinical conditions such as uterine fibroids and adenomyosis, approximately 50% of the cases remain unexplained (Rees, 1987; Davis and Sparzak, 2020) and current treatment options often compromise fertility either temporarily or permanently. We and others have previously demonstrated that vascular structure and maturation is anomalous in the endometrium of women with HMB (Abberton et al., 1999a,b; Hurskainen et al., 1999; Rogers and Abberton, 2003; Biswas Shivhare et al., 2014, 2018).

Recurrent pregnancy loss is defined as two or more consecutive pregnancy losses; many cases are idiopathic (Quenby and Farquharson, 1993; Rai and Regan, 2006), although an altered endometrial environment may be a contributory factor to these idiopathic cases (Brosens and Gellersen, 2010). We and others have shown that women with a history of RPL have increased endometrial blood vessel maturity and blood flow (demonstrated by uterine artery Doppler) (Quenby et al., 2009; El-Azzamy et al., 2018). Embryo implantation occurs in a low oxygen environment (2–3% O_2_) (Yedwab et al., 1976; Rodesch et al., 1992), with oxygen levels in the intervillous space rising to 6–8% O_2_ between 10 and 12 weeks gestation (Jauniaux et al., 2000). Inappropriate maternal blood flow to the intervillous space may be underpinned by several different pathologies and has been proposed to be a final common pathway in both sporadic miscarriage and RPL (Jauniaux et al., 2000). Therefore, we propose that the described changes in the endometrial vasculature in women with a history of RPL may lead to excess oxygen at the early implantation site, resulting in oxidative stress damage to the developing embryo and placenta.

The carefully timed cyclic features of angiogenesis and remodeling of monthly endometrial growth and regression results from a complex network of interactions between hormones and pro- or anti-angiogenic growth factors (Rogers et al., 2009). Although estrogen and progesterone are the primary regulators of endometrial angiogenesis (Iruela-Arispe et al., 1999; Hague et al., 2002), their action is indirectly mediated by several growth factors expressed by the diverse cell population in the endometrium (Hapangama et al., 2015; Kamal et al., 2016). The endometrium is a rich source of angiogenic growth factors (AGFs), which show temporal changes in expression patterns across the menstrual cycle (Lash et al., 2012).

TGFβ1 is secreted as a latent form, its activation and consequent bioavailability is tightly regulated in a multistep process which includes secretion, storage and interaction with ECM components, release from ECM and finally activation prior to receptor binding (ten Dijke and Arthur, 2007). The inactive TGFβ peptide consists of a TGFβ dimer, covalently bound to the latency-associated peptide (LAP) forming the small latent complex (SLC), which can subsequently covalently attach to the large latent TGFβ-binding protein (LTBP1) to form the large latent complex (LLC) (Kanzaki et al., 1990; Saharinen et al., 1996; Maroni and Davis, 2011). The LLC binds to microfibrils and ECM via the LTBP. The degradation of these microfibrils via inflammatory proteolytic enzymes like elastase displaces the LLC from ECM, prior to TGFβ activation (Chaudhry et al., 2007; ten Dijke and Arthur, 2007). Following displacement from ECM, the mature TGFβ dimer is released from the LAP and activated by matrix metalloproteases 2 and 9 (MMP2, MMP9), thrombospondin-1 (THBS1) or integrins in a cell-specific manner, prior to binding to their signaling receptors (Crawford et al., 1998; Annes et al., 2003; Sheppard, 2005; ten Dijke and Arthur, 2007). Active TGFβ dimer signaling is mediated via two pairs of transmembrane receptor serine/threonine kinases knows as the TGFβ type I (TGFβRI) and TGFβ type II (TGFβRII) and/or accessory receptors TGFβRIII (betaglycan) and endoglin (on ECs). One TGFβRII and two distinct TGFβRI receptors; endothelium restricted activin receptor-like kinase (ALK)1 and widely expressed ALK5, are indicated in the TGFβ signaling pathway. Active TGFβ binds to TGFβRII, which recruits, phosphorylates and activates TGFβRI (ALK5), which in turn phosphorylates Smad transcription factors Smad2 and 3, which in turn interact either with SMAD4 to regulate targeted gene expression or with Smad7 resulting in inhibition of this cascade (ten Dijke and Arthur, 2007; Massagué et al., 2008). One of the functions of TGFβ1, is regulation of vascular smooth muscle cell (VSMC) recruitment as well as phenotypic switching from a synthetic to more contractile cell type, which is associated with more mature blood vessels (Lebrin et al., 2005). TGFβ1 and its receptors are expressed in the endometrium across the menstrual cycle, and TGFβ1 is also increased in RPL, a condition associated with increased vascular maturation (Lash et al., 2012). Expression of TGFβ1 has previously been shown to be either unchanged (Abberton et al., 1999b) or reduced in the endometrium of women with HMB (Maybin et al., 2017).

We, and others, have previously demonstrated altered endometrial vascular development in women with HMB and RPL (Abberton et al., 1999b; Quenby et al., 2009; Biswas Shivhare et al., 2014, 2018). We have also shown that TGFβ1 immunoreactivity is increased in the endometrium of RPL (Lash et al., 2012). We therefore hypothesized that expression of TGFβ1, or its receptors, is reduced in the endometrium of women with HMB. We also aimed to further explore the expression of members of the TGFβ1 activation and signaling cascade in endometrium derived from women with a history of RPL and HMB.

## Materials and Methods

### Samples

Endometrial biopsies were obtained with informed consent from women undergoing hysterectomy at the Royal Victoria Infirmary, Newcastle upon Tyne, United Kingdom; Liverpool Women’s Hospital, Liverpool, United Kingdom; or Guangzhou Women and Children’s Medical Center (GWCMC), Guangzhou, China. HMB was defined as excessive menstrual blood loss which interferes with the woman’s physical, emotional, social, and material quality of life, and which can occur alone or in combination with other symptoms (NICE, 2018). In line with the hospital guidelines, empirical blood-loss measurements were not taken, and all cases were idiopathic. All women in the HMB group had unexplained disease, with no known underlying pathologies. RPL was defined as 2 or more consecutive miscarriages with no known causes after extensive examination. The control group was fertile women without any uterine pathology potentially associated with known endometrial abnormality (e.g., endometriosis, adenomyosis, leiomyomata, heavy irregular bleeding, or HMB) undergoing hysterectomy due to prolapse, cystocele, rectocele, urinary, or stress incontinence. Any women who had received hormone treatment within 3 months prior to the operation were excluded from the study. The menstrual cycle phase was staged according to standard morphological criteria (Noyes et al., 1975) by specialist gynecology histopathologists.

Endometrial biopsies (*N* = 5, in each of proliferative-early secretory (P/ES), and mid-late secretory (MS/LS) phases) were obtained from both control and women with HMB. Pipelle biopsies were taken from women with RPL at day 7–9 after the luteinizing hormone (LH) surge as previously described (Lash et al., 2012). The biopsies were fixed in 10% neutral buffered formalin for 24–48 h, routinely processed and embedded in paraffin wax, and 3 μm serial sections were cut for immunohistochemistry. The study was approved by Newcastle and North Tyneside Research Ethics Committee (Ref: 10/H0906/71), Liverpool Adult Ethics Committee (Ref: 09/H1005/55), and the GWCMC Ethics Committee (Ref: 2016021633). Patient demographics are shown in Table 1.

**TABLE 1:** Patient information for control, heavy menstrual bleeding and recurrent pregnancy loss groups [Mean (range)].

Demographic criteria	Control (*n* = 10)	Heavy menstrual bleeding (*n* = 10)	Recurrent pregnancy loss (*n* = 5)
Age (years)	41.1 (28–51)	41.8 (29–51)	38 (29–44)
Parity	2.9 (0–5)	2.2 (0–6)	0 (0–5)
Number of previous miscarriages	0 (0–1)	0 (0–1)	4 (3–7)

Endometrial biopsies for explant culture were obtained from women undergoing hysterectomy at the Royal Victoria Infirmary, Newcastle upon Tyne. Following surgery, the hysterectomy specimen was inspected by a clinical pathologist who excluded any suspicion of significant endometrial or cervical pathology and excised a block of endometrium or myometrium with overlying endometrium measuring approximately 2 × 2 cm. In the research laboratory the biopsy was further dissected into five approximately equal sections, placed into separate wells of a 48-well plate and cultured in control medium [DMEM-F12 containing 10% FBS, penicillin/streptomycin, L-glutamine (Life Technologies, Paisley, United Kingdom)], TGFβ1 (1 ng/ml, 10 ng/ml; Peprotech EC Ltd., London, United Kingdom) or anti-TGFβ1 antibody (10 ng/ml, AbCam) for 72 h, with treatment medium being changed after every 24 h. Controls contained 2 μM citric acid diluted in the appropriate culture medium. After 72 h, the tissue sections were removed from the plate, immediately fixed in neutral buffered formalin in five separately labeled containers and FFPE sections prepared for immunohistochemistry analysis.

### Immunohistochemistry

Paraffin sections (3 μm) were dewaxed in xylene, rehydrated through descending concentrations of alcohol and incubated in 1% H^2^O^2^ in water for 10 min to block endogenous peroxidase activity. All washes were performed in 0.15M Tris-buffered 0.05M saline, pH 7.6 (TBS). Immunostaining was performed as previously described in detail (Schiessl et al., 2009). Antibodies were detected using an avidin-biotin-peroxidase technique (Vectastain Elite ABC kit; Vector Laboratories, Peterborough, United Kingdom). Details of source and dilution for all antibodies are provided in Supplementary Table 1. The reaction was developed for 1–2 min with 3,3′-diaminobenzidine (DAB; Sigma Chemical Co. Dorset, United Kingdom) containing 0.01% H_2_O_2_ to give a brown reaction product. Following this, sections were lightly counterstained with Mayer’s hematoxylin for 30 s, dehydrated, cleared in xylene and mounted with DPX synthetic resin (Raymond A. Lamb Ltd., London, United Kingdom). Positive and negative (replacement of the primary antibody by appropriate non-immune serum) controls were performed for all antibodies and samples.

### Quantitative Image Analysis

#### Angiogenic Growth Factor (AGF) Expression in the Endometrium

Sections were examined using a Nikon Eclipse 80i microscope with a 20x objective and 10x eyepiece. AGF expression was assessed in the endometrial vessels, stroma, and glands (*N* = 5) in each of P/ES, MS/LS from both controls and women with HMB, and MS/LS from women with RPL by an observer, who was blinded to the origin of the sample using a modified “Quickscore” method (Schiessl et al., 2009). The whole of each section was assessed and the proportion of cells (1 = 0–25%, 2 > 25–50%, 3 > 50–75%, 4 > 75–100%) staining at a particular intensity (0 = negative, 1 = weak, 2 = moderate, 3 = strong) was taken into account. The percentage and intensity scores were then multiplied and summed to give a possible score range of 0–12. Vessels were identified by their smooth profile and by the surrounding layer(s) of smooth muscle cells. Only vessels with a visible lumen were included in the analysis.

#### VSMC Differentiation Marker Expression and ECM Component Expression in Cultured Endometrium

Sections were examined using a Nikon Eclipse 80i microscope with a 20x objective and 10x eyepiece. Expression of VSMC differentiation markers, EC, and ECM components was assessed in vessels in the entire tissue biopsies (*N* = 3 endometrium) in the proliferative phase from controls by an observer, who was blinded to the origin of the sample. Vessels were identified by their smooth profile and by the surrounding layer(s) of smooth muscle cells. Only vessels with a visible lumen were included in the analysis. Expression of VSMC differentiation markers and vascular ECM components was assessed by counting manually the percentage of vessels positively stained for each marker compared with the total number of vessels in the entire tissue as identified by CD31 + staining. VSMC differentiation marker immunostaining intensity was assessed by modified “Quickscore” as described above.

### Isolation of Endothelial Cells and Vascular Smooth Muscle Cells From Saphenous Vein

Segments of saphenous vein (SV), obtained during coronary bypass operation, ≥2 cm in length were used for isolation of ECs and VSMCs. Veins were flushed with sterile PBS (pH 7.4) to remove red blood cells, one end constricted and filled with warmed endothelial cell digestion medium (0.1% Type I collagenase in PBS; Sigma), the other end was then constricted and incubated for 15 min at 37°C. The vein segment was then flushed with 3–4 ml PBS (pH 7.4), the medium collected, centrifuged (200 g for 5 min) and the resultant cell pellet re-suspended in EC culture medium (EC Growth Kit-BBE in vascular cell basal medium; LGC Standards Ltd, Teddington, United Kingdom), plated into one well of a 6-well plate and cultured at 37°C. A second 40 min incubation in EC digestion medium was performed and the resultant cell pellet also re-suspended in EC culture medium and plated. A final 50 min incubation in VSMC digestion medium [0.1% Type I collagenase, 0.02% elastase, 0.02% DNase in PBS (pH7.4); Sigma] was performed, the resultant cell pellet re-suspended in VSMC culture medium (Ham’s F12K containing 10% FBS, 30 μg/ml EC growth supplement, 50 μg/ml ascorbic acid, 1% HEPES, 2.3 mg/ml TES, 1% insulin/transferrin/sodium selenite, 1% amphotericin, 1% penicillin/streptomycin; LGC Standards or Sigma), plated in one well of a 6-well plate and incubated at 37°C. After 24 h, medium in each well was replaced with 2 ml appropriate EC or VSMC medium and incubated at 37°C until 90% confluence was reached. Isolated cells were characterized by immunocytochemistry for Factor VIII related antigen (F8RA), CD31 and CD34 (ECs), and alpha-smooth muscle actin (αSMA), h-caldesmon, and myosin heavy chain (VSMCs). Cells that were >99% pure and were between passages 3 and 5 were used for further experiments.

### EC:VSMC Association Assay

ECs and VSMCs were incubated at 37°C, 5% CO_2_ for 30 min in cell tracker CFSE (5 mM stock diluted 1:5000 in EC basal medium; Invitrogen) and CMRA (10 mM stock diluted 1:2000 in VSMC basal medium; Invitrogen), respectively. EC (6 × 10^3^ cells/well) were plated into 8-well chambers slides, 4 wells of which had been precoated with 5 μl growth factor reduced Matrigel (BD) and incubated for 4.5 h at 37°C at which point VSMCs (6 × 10^3^ cells/well) in cell media (0, 1, 10 ng/ml TGFβ1) were added. Following a further 1 h incubation, time-lapse live imaging was performed on a Nikon Eclipse-i laser-scanning confocal microscope, equipped with 37°C incubator and 5% CO_2_ supply, using 20x oil objective, 10x eyepiece and excitation wavelengths of 492 nm (CFSE) and 548 nm (CMRA). Live imaging was carried out for up to 10 h and images captured every 15 min.

In order to study EC:VSMC interaction on matrigel, the following parameters were included: (a) total number of honeycomb (or tubule) structures with ≥ 3 arms, (b) the total number of nodes, (c) arm length, (d) the number of complete honeycombs (with an enclosed area), (e) the perimeter of the complete honeycombs, and finally (f) the percentage of VSMC coverage of the complete honeycombs determined as described below.

The digital time-lapse movies were analyzed using the open-source ImageJ software package in combination with the “cell counter” plugin to manually count the total number of honeycombs, number of nodes, complete honeycombs and measure the perimeter of three randomly chosen complete honeycombs at *t* = 10 h and the length of 5 randomly chosen arms at *t* = 10 h. The percentage of VSMC coverage was analyzed by measuring the total length of one complete honeycomb covered with VSMCs with respect to the EC perimeter length of the honeycomb.

### AGF Secretion

Saphenous vein-derived ECs and VSMCs were separately cultured on 6-well plates and treated with TGFβ1 (0, 1, and 10 ng/ml) for 24 h. The resulting conditioned medium was harvested and used to determine the relative levels of AGFs by ELISA (VEGF-C, Ang-1, TGF-β1; R&D Systems Ltd) or Quantibody multiplex array (Angiogenin, Ang-2, EGF, bFGF, HB-EGF, HGF, Leptin, PDGF-BB, PlGF, VEGF-A; Raybiotech, Norcross, United States) according to the manufacturers’ instructions. Each experiment was performed on three separate occasions.

### Statistical Analysis

Statistical analyses were performed using SPSS version 15.0 (SPSS Inc.). Data are presented as means ± standard error of mean (SEM) and differences were considered statistically significant at *P* ≤ 0.05. For comparison of three or more groups, Kruskal–Wallis or ANOVA with *post hoc* tests were used for non-parametric or parametric data as appropriate. For comparison of 2 groups Mann–Whitney U or Students *t* test were used for non-parametric or parametric data as appropriate.

## Results

### Factors Involved in TGFβ1 Activation

**FIGURE 1:**
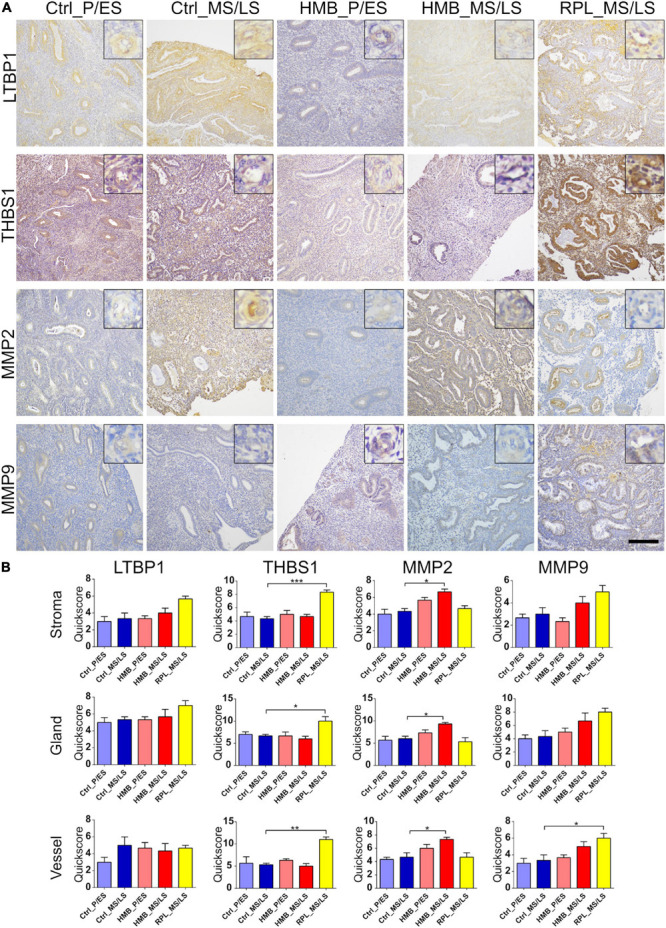
**(A)** Representative photomicrographs of immunostaining for large latent TGFβ-binding protein (LTBP1), thrombospondin (THBS1), matrix metalloproteinase (MMP)2 and MMP9 in proliferative/early secretory (P/ES), mid/late secretory (MS/LS) phase of the menstrual cycle in control and women with heavy menstrual bleeding (HMB), and MS/LS phase of women with recurrent pregnancy loss (RPL). Scale bar = 200 μm. Insets show enlarged magnification of one endometrial blood vessel. **(B)** Histograms showing quickscore for LTBP1, THBS1, MMP2, and MMP9 in endometrial stromal cells, glandular epithelium and blood vessels (EC + VSMC) *n* = 5 each group. * = *P* < 0.05, ** = *P* < 0.01, *** = *P* < 0.001.

There was weak to moderate immunoreactivity for LTBP1 in the stroma, glandular epithelium and vessels of the endometrium that did not alter across the menstrual cycle on in cases of HMB or RPL (Figures 1A,B). THBS1 showed moderate immunoreactivity in the endometrial stroma, glandular epithelium and vessels that did not alter across the menstrual cycle in control women or in women with HMB (Figures 1A,B). However, THBS1 immunoreactivity was increased in the stroma (*P* < 0.001), glandular epithelium (*P* < 0.05) and vessels (*P* < 0.01) of women with RPL compared with control women in the MS/LS phase of the menstrual cycle (Figures 1A,B). MMP2 showed weak to moderate immunoreactivity in the endometrial stroma, glandular epithelium, and vessels that did not alter with the menstrual cycle in control women or in women with RPL (Figures 1A,B). In MS/LS endometrium of women with HMB, MMP2 immunoreactivity was increased compared with controls (Stroma *P* < 0.05, glandular epithelium *P* < 0.05, vessels *P* < 0.05) (Figures 1A,B). Immunoreactivity for MMP9 was weak in all sections of the endometrium examined and did not alter with the menstrual cycle in control women or in women with HMB (Figures 1A,B); however, in comparison, women with RPL demonstrated an increase in MMP9 immunoreactivity in the endometrial vessels (*P* < 0.05) (Figures 1A,B).

### TGFβ1 and Receptors

**FIGURE 2:**
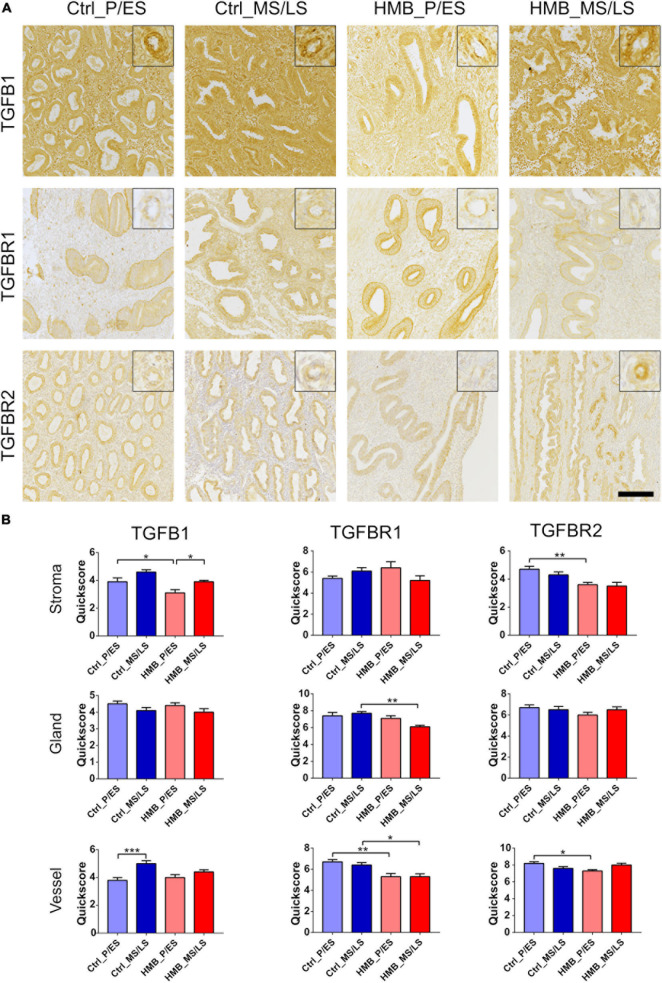
**(A)** Representative photomicrographs of immunostaining for TGFβ1, TGFβRI, and TGFβRII in proliferative/early secretory (P/ES), mid/late secretory (MS/LS) phase of the menstrual cycle in control and women with heavy menstrual bleeding (HMB), and MS/LS phase of women with recurrent pregnancy loss (RPL). Scale bar = 200μm. Insets show enlarged magnification of one endometrial blood vessel. **(B)** Histograms showing quickscore for TGFβ1, TGFβRI, and TGFβRII in endometrial stromal cells, glandular epithelium and blood vessels (EC + VSMC) *n* = 5 each group. * = *P* < 0.05, ** = *P* < 0.01, *** = *P* < 0.001.

We have previously reported that immunoreactivity for TGFβ1 is increased in the endometrium of women with RPL, while TGFβRI and TGFβRII are decreased (Lash et al., 2012). In the current study immunoreactivity for TGFβ1 was weak to moderate and was increased in the vessels of control women in the MS/LS phase of the menstrual cycle compared to the P/ES phase (*P* < 0.001) (Figures 2A,B). Stromal immunoreactivity was reduced in the P/ES phase of women with HMB compared with controls (*P* < 0.05), and in women with HMB was increased in the MS/LS phase compared with the P/ES phase (*P* < 0.05) (Figures 2A,B). Immunoreactivity for TGFβRI and TGFβRII was also weak to moderate and did not alter with the menstrual cycle in control women or women with HMB (Figures 2A,B). Vascular immunoreactivity for TGFβRI was reduced in women with HMB in both the P/ES (*P* < 0.01) and MS/LS (*P* < 0.05) phases of the menstrual cycle compared with controls. Glandular immunoreactivity was also reduced in the MS/LS phase of HMB women compared with controls (*P* < 0.01) (Figures 2A,B). In addition, stromal (*P* < 0.01) and vascular (*P* < 0.05) immunoreactivity for TGFβRII was reduced in P/ES phase of the menstrual cycle of women with HMB compared with controls (Figures 2A,B).

**FIGURE 3:**
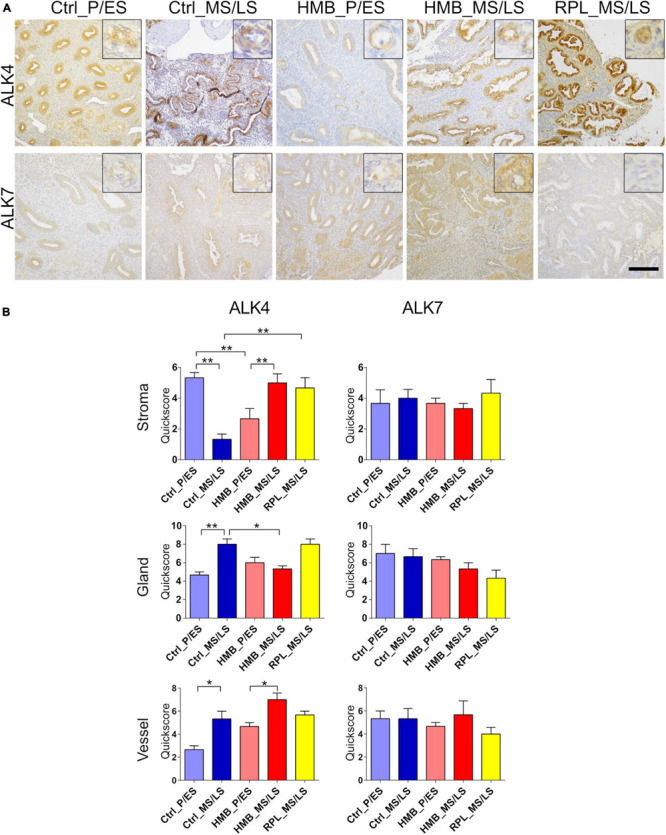
**(A)** Representative photomicrographs of immunostaining for activin-like receptor (ALK)4 and ALK7 in proliferative/early secretory (P/ES), mid/late secretory (MS/LS) phase of the menstrual cycle in control and women with heavy menstrual bleeding (HMB), and MS/LS phase of women with recurrent pregnancy loss (RPL). Scale bar = 200 μm. Insets show enlarged magnification of one endometrial blood vessel. **(B)** Histograms showing quickscore for ALK4 and ALK7 in endometrial stromal cells, glandular epithelium and blood vessels (EC + VSMC) *n* = 5 each group. * = *P* < 0.05, ** = *P* < 0.01.

Immunoreactivity for ALK4 was weak to moderate in all cell types and was reduced in the stroma of control women in the MS/LS phase of the menstrual cycle compared to the P/ES phase (*P* < 0.01), but was increased in both the glandular epithelium (*P* < 0.01) and vessels (*P* < 0.05) (Figures 3A,B). In addition, ALK4 was increased in stroma (*P* < 0.01) and vessels (*P* < 0.05) in the MS/LS phase of the menstrual cycle of women with HMB compared with the P/ES phase. In the P/ES phase of the cycle of women with HMB, ALK4 immunoreactivity in the stroma was reduced compared with the control samples from the same cycle phase (*P* < 0.01). In the MS/LS of women with HMB, ALK4 immunoreactivity in the glandular epithelium was reduced compared to controls (*P* < 0.05). In the MS/LS of women with RPL, ALK4 immunoreactivity in the stroma was increased compared to controls (*P* < 0.01). ALK7 immunoreactivity was weak to moderate in the stroma, glandular epithelium and vessels and did not alter across the menstrual cycle of controls, or in women with HMB or RPL (Figures 3A,B).

**FIGURE 4:**
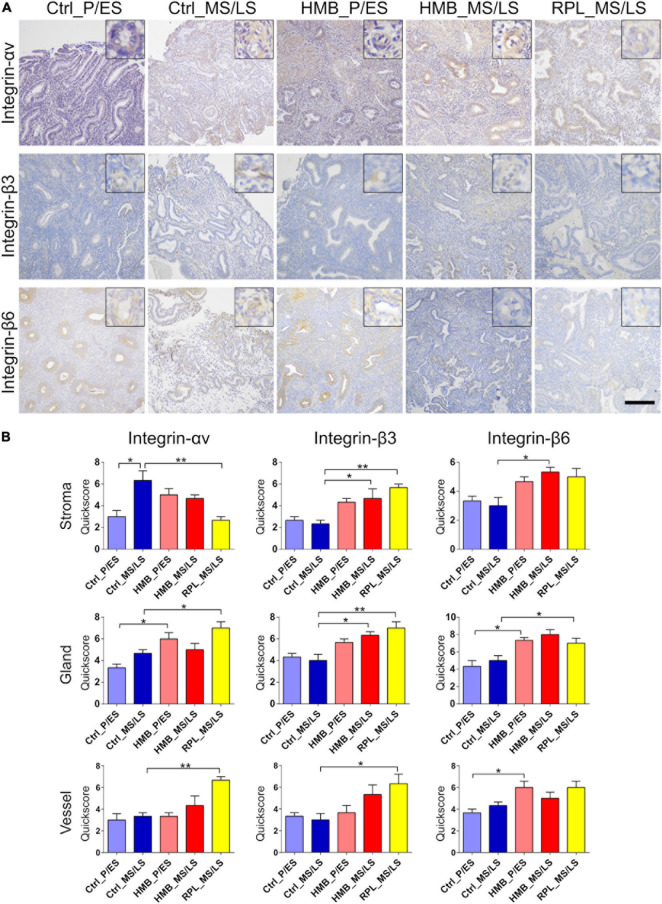
**(A)** Representative photomicrographs of immunostaining for integrins αv, β3, and β6 in proliferative/early secretory (P/ES), mid/late secretory (MS/LS) phase of the menstrual cycle in control and women with heavy menstrual bleeding (HMB), and MS/LS phase of women with recurrent pregnancy loss (RPL). Scale bar = 200 μm. Insets show enlarged magnification of one endometrial blood vessel. **(B)** Histograms showing quickscore for integrins αv, β3, and β6 in endometrial stromal cells, glandular epithelium and blood vessels (EC + VSMC) *n* = 5 each group. * = *P* < 0.05, ** = *P* < 0.01.

Immunoreactivity for integrin αv was weak in stroma, glandular epithelium, and vessels, which was increased in the stroma in the MS/LS phase of controls compared with the P/ES phase (*P* < 0.05) (Figures 4A,B). In the P/ES phase glandular epithelium of women with HMB, integrin αv immunoreactivity was increased compared to controls (*P* < 0.05). In RPL, integrin αv immunoreactivity in stroma was decreased (*P* < 0.01), but increased in glandular epithelium (*P* < 0.05) and vessels (*P* < 0.01) compared to controls. Integrin β3 immunoreactivity was weak in stroma, glandular epithelium, and vessels that did not alter across the menstrual cycle of controls (Figures 4A,B). In women with HMB and RPL MS/LS, immunoreactivity of integrin β3 in stroma (HMB *P* < 0.05, RPL *P* < 0.01) and glandular epithelium (HMB *P* < 0.05, RPL *P* < 0.01) was increased compared to controls; in RPL vessel (*P* < 0.05), immunoreactivity was also increased compared to controls. Integrin β6 immunoreactivity in stroma, glandular epithelium, and vessels was weak and did not change across the menstrual cycle (Figures 4A,B). In the P/ES phase of the menstrual cycle in women with HMB immunoreactivity for integrin β6 in glandular epithelium (*P* < 0.05) and vessels (*P* < 0.05) was increased compared with controls, while stromal immunoreactivity was increased in the MS/LS phase of the menstrual cycle (*P* < 0.05) (Figures 4A,B). In women with RPL glandular epithelial immunoreactivity of integrin β6 was increased compared to controls (*P* < 0.05).

### TGFβ1 Signaling Pathway

**FIGURE 5:**
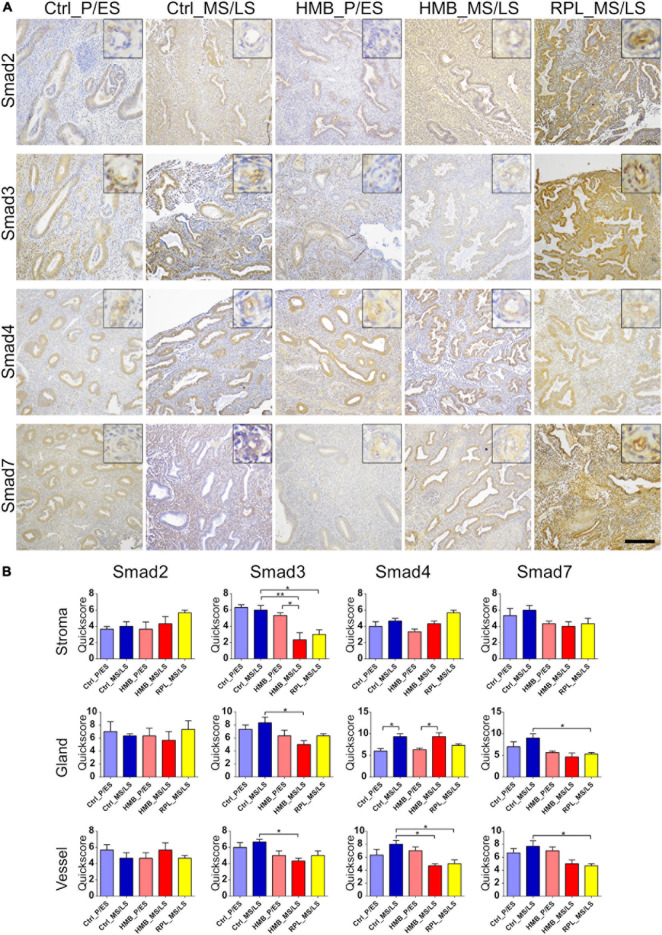
**(A)** Representative photomicrographs of immunostaining for Smad2, Smad3, Smad4, and Smad7 in proliferative/early secretory (P/ES), mid/late secretory (MS/LS) phase of the menstrual cycle in control and women with heavy menstrual bleeding (HMB), and MS/LS phase of women with recurrent pregnancy loss (RPL). Scale bar = 200 μm. Insets show enlarged magnification of one endometrial blood vessel. **(B)** Histograms showing quickscore for Smad2, Smad3, Smad4, and Smad7 in endometrial stromal cells, glandular epithelium, and blood vessels (EC + VSMC) *n* = 5 each group. * = *P* < 0.05, ** = *P* < 0.01.

Immunoreactivity for Smad2 was weak to moderate in endometrial stroma, glandular epithelium and vessels, which did not alter across the menstrual cycle of controls or in women with HMB or RPL (Figures 5A,B). Moderate immunostaining for Smad3 was observed in stroma, glandular epithelium and vessels, and did not alter across the menstrual cycle in controls (Figures 5A,B). Smad3 immunoreactivity was reduced in MS/LS phase stroma (*P* < 0.01), glandular epithelium (*P* < 0.05) and vessels (*P* < 0.05) in women with HMB compared to controls, stromal Smad3 was also reduced in women with RPL (*P* < 0.05) (Figures 5A,B). Smad4 immunoreactivity was weak to moderate in stroma, glandular epithelium, and vessels and increased in glandular epithelium of controls (*P* < 0.05) and HMB (*P* < 0.05) in MS/LS compared with P/ES phase endometrium (Figures 5A,B). Vessel immunoreactivity of Smad4 was reduced in MS/LS phase in both women with RPL (*P* < 0.05) and HMB (*P* < 0.05) compared to controls (Figures 5A,B). Moderate immunoreactivity for Smad7 was observed in stroma, glandular epithelium, and vessels and did not alter across the menstrual cycle of controls or in women with HMB (Figures 5A,B). Immunoreactivity for Smad7 was reduced in both endometrial glandular epithelium (*P* < 0.05) and vessels (*P* < 0.05) of women with RPL compared with controls (Figures 5A,B).

### Effect of TGFβ1 on Endometrial Vascular Development

**FIGURE 6:**
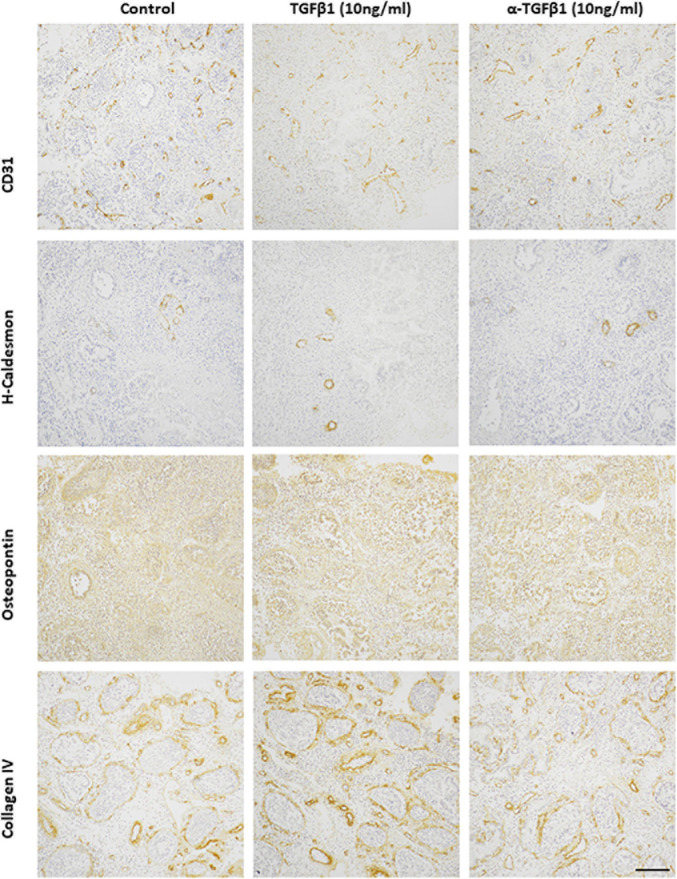
Representative photomicrographs of TGFβ1 (10 ng/ml) and anti-TGFβ1 (10 ng/ml) treated proliferative endometrium explants immunostained for CD31, h-caldesmon, osteopontin, collagen IV. Scale bar = 200 μm.

To determine whether TGFβ1 plays a role in endometrial vascular development small endometrial biopsies were cultured in TGFβ1 or anti-TGFβ1 and assessed by immunohistochemistry for the total number of CD31^+^ vessels, the percentage of CD31^+^ vessels that also expressed VSMC markers or ECM components, and the intensity of immunoreactivity of those markers. Representative photomicrographs are shown in Figure 6.

The total number of CD31^+^ endometrial vessels did not alter after treatment with TGFβ1 (10 ng/ml) or anti-TGFβ1 (10 ng/ml) (control 7.3 ± 2.7; TGFβ1 6.9 ± 3.6; anti-TGFβ1 6.6 ± 4.3). Around 30% CD31^+^ endometrial vessels also expressed h-caldesmon in the untreated control group. This was reduced when treated with either TGFβ1 (∼20%) or anti-TGFβ1 (∼12%) compared with the untreated group. The immunoreactivity of h-caldesmon did not alter with treatment (control 2.0 ± 0; TGFβ1 3.0 ± 0; anti-TGFβ1 4.0 ± 1.0). Around 10% CD31^+^ endometrial vessels also expressed smoothelin in the untreated control group, that was reduced after treatment with TGFβ1 (< 5%) or anti-TGFβ1 (< 5%). Smoothelin immunoreactivity intensity was not altered by treatment (control 1.0 ± 1.0; TGFβ1 1.0 ± 1.0; anti-TGFβ1 2.0 ± 2.0). Around 15% CD31^+^ endometrial vessels also expressed calponin, which was reduced after treatment with TGFβ1 (< 5%) or anti-TGFβ1 (< 5%). However, the immunoreactivity intensity for calponin was not altered (control 2.5 ± 0.5; TGFβ1 2.5 ± 0.5; anti-TGFβ1 2.0 ± 1.0). In the endometrial explants, all of the CD31^+^ vessels expressed collagen IV and osteopontin in the untreated control group, which was unaltered after treatment with TGFβ1 (100%) or anti-TGFβ1 (100%). The immunoreactivity intensity was also unaltered by treatment (collagen IV: control 9.0 ± 1.0; TGFβ1 9.5 ± 1.5; anti-TGFβ1 10.5 ± 1.5; osteopontin: control 8.0 ± 0; TGFβ1 7.5 ± 0.5; anti-TGFβ1 8.0 ± 1.0).

### Effect of TGFβ1 on EC:VSMC Association *in vitro*

**FIGURE 7:**
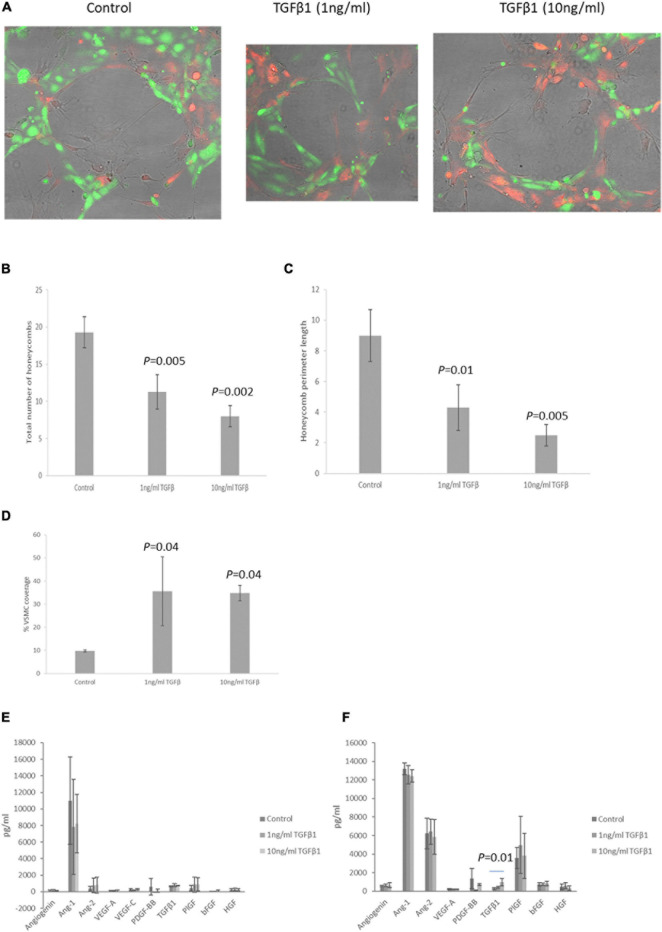
**(A)** Representative photomicrographs of EC tubules (green) covered by VSMCs (red) in control and TGFβ1 (1 and 10 ng/ml) treated wells (10 h). **(B–D)** Graphical representation of endothelial cell honeycomb number **(B)**, honeycomb perimeter length **(C)** and percentage VSMC coverage of the EC tubules **(D)** in the VSMC:EC association assay after treatment with TGFβ1 for 10 h. **(E,F)** EC **(E)** and VSMC **(F)** secretion of a range of angiogenic growth factors after TGFβ1 treatment. *N* = 5 all conditions. Data are shown as mean ± SD.

To determine how TGFβ1 effects the association of EC and VSMCs, we developed an *in vitro* assay extension of the commonly used EC tubule formation assay (Figure 7A). In addition to assessing the total number of honeycombs formed and the average length of the perimeter of each honeycomb, the % of honeycomb length covered by VSMCs was also determined. The total number of nodes and arm lengths were also assessed but this did not alter with treatment (data not shown). Treatment with TGFβ1 reduced the total number of honeycombs after 10 h (1 ng/ml, *P* = 0.005; 10 ng/ml *P* = 0.002) (Figure 7B), and the perimeter length (1 ng/ml, *P* = 0.01; 10 ng/ml, *P* = 0.005) (Figure 7C). However, it also increased the percentage of EC honeycombs covered by VSMCs (1 ng/ml, *P* = 0.04; 10 ng/ml, *P* = 0.04) (Figure 7D).

To determine the potential mechanism by which TGFβ1 might increase EC:VSMC association secretion of a range of angiogenic growth factors by EC or VSMC after treatment with TGFβ1 was assessed by multiplex assay. Neither EC or VSMC secreted detectable levels of leptin, EGF or HB-EGF, and VSMC did not have detectable levels of VEGF-C. Treatment of EC with TGFβ1 did not alter secretion levels of any of the proteins investigated (Figure 7E). Treatment of VSMC with TGFβ1 only increased the secretion of TGFβ1 itself (10 ng/ml, *P* = 0.01, Figure 7F).

## Discussion

Both HMB and RPL are associated with alterations in endometrial vascular development. In the current study, we demonstrate dysregulation of TGFβ1 and associated activation and signaling pathway proteins in the endometrium of women with HMB or RPL compared with normal controls. TGFβ1 is one of the key growth factors that drives VSMC maturation, and using an *in vitro* EC:VSMC association assay, we demonstrate an increased association of VSMCs with pre-existing EC tubules after treatment with TGFβ1. This is one of the most comprehensive immunohistochemical studies of TGFβ1 binding and signaling pathway components in the normal and pathological endometrium and will give us insights into the importance of this growth factor in endometrial regeneration and pathology.

Angiogenesis and vascular development take place in the endometrium across the menstrual cycle through elongation of vascular stubs in the endometrium basalis, which were left after menstrual shedding of the endometrium functionalis (Girling and Rogers, 2005, 2009). While the process of blood vessel growth and development in endometrium remains under the master control of steroid hormones, angiogenic growth factors such as TGFβ1 and its receptors may directly regulate EC proliferation and/or apoptosis, interaction with and integrity of ECM components, as well as recruitment and maturation of VSMCs, which ultimately results in vascular development and maturation (Dickson et al., 1995; Pepper, 1997; Massague et al., 2000; Tabibzadeh, 2002; Distler et al., 2003). The majority of TGFβ1 is found as a latent form bound to latency associated proteins and then often also held within the ECM close to the cell membrane by different integrins. When required, it can be activated by proteases or thrombospondin and bind to TGFβRII, which recruits TGFβRI or the alternative receptors ALK4 and ALK7, thereby setting up the Smad signaling pathway involving phosphorylation of Smads2, 3, 4, and 7, with eventual nuclear translocation of p-Smad4 and transcription regulation. Therefore, alteration in the expression of any of these factors will lead to aberrant vascular development, dependent on the balance of direction of change for all these pathway components.

**FIGURE 8:**
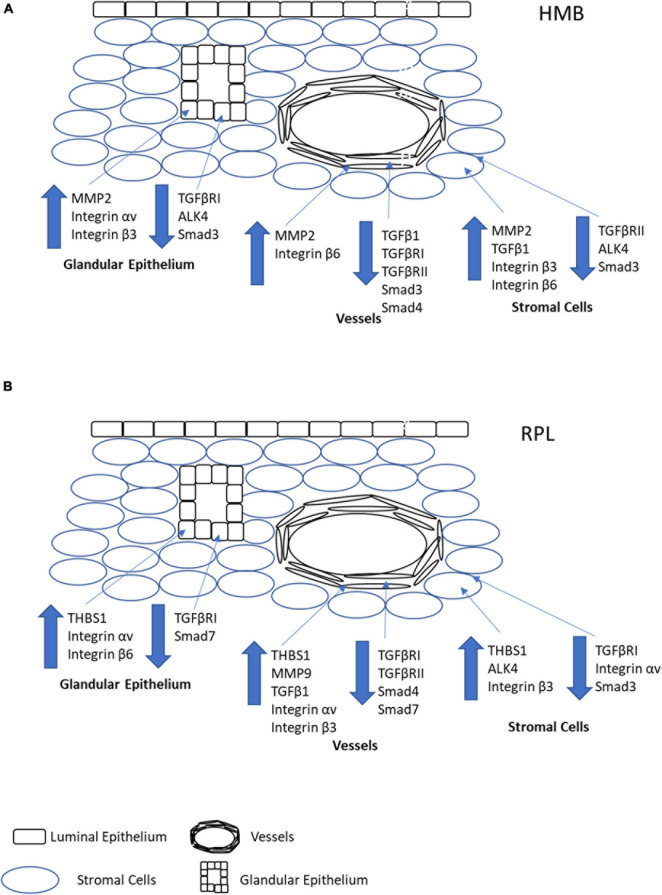
Pictorial representation of the overall changes in immunoreactivity stromal cells, glandular epithelium, and vessels in the endometrium of women with HMB **(A)** or RPL **(B)**. Note that for simplicity, immune cells have not been depicted in the stroma, and only factors which demonstrated significant differences in immunoreactivity have been shown.

In HMB, we and others have shown a failure in vascular maturation in the mid-late secretory phase of the menstrual cycle (Abberton et al., 1999b; Biswas Shivhare et al., 2014; Biswas Shivhare et al., 2018). In the current study, we demonstrate that in women with HMB, there was no change in the immunoreactivity for LTBP1, THBS1, or MMP9 in any of the cell types investigated. However, MMP2 immunoreactivity in stromal cells, glandular epithelium, and vessels was increased suggesting a potential increase in bioactive TGFβ1. However, there was a decrease in immunoreactivity in TGFβ1 (stromal cells), TGFβRI (glandular epithelium and vessels), TGFβRII (stromal cells and vessels), and ALK4 (stromal cells and glandular epithelium), which together suggest lowered levels of TGFβ1 and availability of both classical and alternative receptors. In particular, lowered stromal cell TGFβ1 and reduced vascular immunoreactivity for both TGF receptors would suggest a decrease in bioactivity in the maturing vasculature. In addition, there was an increase in the immunoreactivity of integrins β3 (stromal cells and glandular epithelium) and β6 (stromal cells and vessels), which bind active TGFβ1 making it available for receptor binding. Finally, there was decreased immunoreactivity for Smad3 (all cell types) and Smad4 (vessels), suggesting a decrease in signaling capacity, particularly in the developing vasculature. These data suggest an overall decrease in TGFβ1, subsequent receptor, and signaling capacity predominantly localized to the vasculature and thereby contributing to the decreased vascular maturation observed in women with HMB (Figure 8A). In contrast, Rae et al. (2009) demonstrated a decrease in mRNA for THBS1 in women with HMB compared with controls. *In vitro* studies suggested that the reduced THBS1 was due to an inactivation of cortisol, and both THBS1 and cortisol led to increased tubule formation of ECs (Rae et al., 2009). In addition to its ability to activate latent TGFβ1 in a non-proteolytic manner, THBS1 is also an angiogenesis inhibitor. However, in our experience, HMB is not associated with dysregulated angiogenesis but with a failure of maturation of the endometrial blood vessels, leading to premature and extended bleeding and menstrual shedding that may also be associated with hypoxia due to this failure to develop an adequate vascular environment (Maybin et al., 2018). Two other studies have previously investigated TGFβ1 and associated proteins in the endometrium of HMB with varying results. Abberton et al. (1999b) demonstrated no difference in TGFβ1 immunoreactivity in an immunohistochemical study of endometrium from control women and those with HMB. More recently, Maybin et al. (2017) reported no difference in mRNA levels for TGFβ1, TGFβRI, or TGFβRII in endometrium of women with HMB compared with controls. However, they did demonstrate a decrease in immunoreactivity for TGFβ1 in the stromal cells of women with HMB compared with controls. In addition, they also demonstrated a decrease in Smad2 and Smad3 mRNA and levels of their corresponding phosphoproteins in women with HMB. The findings of the current study are more consistent with those of Maybin et al. (2017), and any differences may be due to differences in methodology, menstrual cycle groupings, and classification of HMB. Maybin et al. (2017) used an empirical definition of HMB with 80 mL menstrual blood loss as the cut off, whereas blood loss was not objectively measured in our study, and we used the current clinical assessment of patient declared HMB; thus, the classification used in the current study was more subjective.

In contrast to HMB, we and others have shown increased endometrial vascular maturation in women with RPL (Quenby et al., 2009; El-Azzamy et al., 2018). We had also previously shown that immunoreactivity for TGFβ1 (vessels) was increased, and both TGFβRI (stromal cells, glandular epithelium, and vessels) and TGFβRII (vessels) decreased in the endometrium of women with RPL compared to controls (Lash et al., 2012). In the current study, we also show an increase in both THBS1 (all cell types) and MMP9 (vessels), suggesting an increase in both the non-proteolytic and proteolytic pathways for TGFβ1 activation in the developing vasculature. In addition, immunoreactivity for the alternative receptor ALK4 (stromal cells) was increased, as were the integrins β3 (stromal cells and vessels) and β6 (glandular epithelium). Immunoreactivity for integrin αv had variable expression, was increased in the glandular epithelium and vessels, but reduced in stromal cells. These data suggest potential increased bioavailability of and binding capacity for TGFβ1. In addition, immunoreactivity for Smad3 (stromal cells), 4 (vessels), and 7 (glandular epithelium and vessels) were all decreased in women with RPL. Taken together, these data suggest an increase in bioactive TGFβ1 in and near the vasculature, although signaling capacity may be reduced. The observed increase in bioactive TGFβ1 likely contributes to the increased vascular maturation observed in women with RPL (Figure 8B).

Using two different *in vitro* models, culture of endometrial explants and a novel EC:VSMC association assay, it was also demonstrated that treatment with TGFβ1 increased recruitment of VSMCs to the endometrial vasculature as well as their differentiation into a more contractile phenotype. In addition, TGFβ1 increased TGFβ1 secretion by VSMCs, but not ECs, and did not alter secretion of any other factor investigated suggesting the existence of a positive regulation loop for vascular TGFβ1 expression. TGFβ1 is the best described growth factor driving the differentiation of the phenotypically plastic VSMCs from the synthetic state to the more mature contractile state. VSMC phenotypic switching is primarily regulated by the master transcription factors mycodelin and KLF4, with mycodelin driving differentiation to a contractile state and KLF4 driving the reverse. TGFβ1 can regulate mycodelin expression either directly via the Smad pathway or via regulation of different miRNA species (Chan et al., 2010; Davis-Dusenbery et al., 2011; Lim et al., 2020).

One limitation of the current study is that is uses semiquantitative immunohistochemical scoring to assess expression levels of the investigated proteins. However, the advantage of this technique is that we can assess different cellular components separately, and as was revealed, changes in the expression patterns were often cell specific, and these results may have been masked by whole tissue Western Blot or qPCR studies (Maclean et al., 2020). The second limitation is the use of subjective measurement criteria for HMB; while these criteria are in line with NICE guidelines (2018) and are in accordance with routine clinical practice, it is possible that some of our patients did not have greater than 80 mL blood loss and that some of our control women did but had not reported HMB symptoms. However, since the women were undergoing a major surgery, a hysterectomy, for their HMB, we believe our HMB group was a clinically suitable group to study.

Taken together, the data presented in the current study suggest that TGFβ1 is a key regulatory pathway for vascular maturation in the endometrium across the menstrual cycle, and when this pathway is dysregulated, it contributes to the altered vascular development observed in conditions such as HMB and RPL. However, we still do not understand how the TGFβ1 pathway is regulated in the endometrium, and studies are currently underway in our laboratories to investigate the role of miRNA in the regulation of this pathway in both HMB and RPL. Manipulation of the TGFβ1 pathway maybe a viable option for the treatment of both HMB and RPL in women who wish to preserve their fertility, especially as the development of TGFβ1 pathway modulators is already being explored for the treatment of metastatic cancer, with the possibility of repurposing of these therapeutics for the treatment of women with HMB and/or RPL, although any treatment in the latter group would need to be pre-pregnancy.

## Data Availability Statement

The raw data supporting the conclusions of this article will be made available by the authors, without undue reservation.

## Ethics Statement

The studies involving human participants were reviewed and approved by the Newcastle and North Tyneside Research Ethics Committee (Ref:10/H0906/71), Liverpool Adult Ethics Committee (Ref: 09/H1005/55), and the GWCMC Ethics Committee (Ref: 2016021633). The patients/participants provided their written informed consent to participate in this study.

## Author Contributions

QL and SS were involved in the study design, data collection, and analysis. DS was involved in the study design and data collection. HH was involved in the data collection. JB played a role in the primary histopathological assessment of all sections and in the study design and contributed toward the critical discussion of the manuscript. BI was involved in the sample collection and preparation. DH contributed toward the sample collection and the critical discussion of the manuscript. GL played a role in the study design, writing, and critical discussion of the manuscript. All authors contributed to the article and approved the submitted version.

## Conflict of Interest

The authors declare that the research was conducted in the absence of any commercial or financial relationships that could be construed as a potential conflict of interest.
