# Supplementary material for: Transforming Growth Factor (TGF) β and Endometrial Vascular Maturation
Source: Front Cell Dev Biol. 2021 Apr 9;9:640065. doi: 10.3389/fcell.2021.640065 (PMC8063037; doi:10.3389/fcell.2021.640065)
Supplement: Supplementary file 1 [file Table_1.docx]

**Supplementary Table 1:** Primary Antibodies used for immunohistochemistry

| **Antibody** | **Dilution** | **Incubation Time** | **Species** | **Clone or Cat#** |
| --- | --- | --- | --- | --- |
| ALK4^1^ | 1/100 | Overnight, 4ºC | Rabbit | ab109300 |
| ALK7^2^ | 1/200 | Overnight, 4ºC | Rabbit | NBP2-15272 |
| Integrin-αv^1^ | 1/500 | Overnight, 4ºC | Rabbit | ab179475 |
| Integrin-β3^1^ | 1/100 | Overnight, 4ºC | Rabbit | ab179473 |
| Integrin-β6^1^ | 1/200 | Overnight, 4ºC | Rabbit | ab233519 |
| LTBP1^3^ | 1/50 | Overnight, 4ºC | Rabbit | GTX81296 |
| MMP2^4^ | 1/200 | Overnight, 4ºC | Mouse | AM1844b |
| MMP9^1^ | 1/200 | Overnight, 4ºC | Rabbit | ab76003 |
| Smad2^5^ | 1/100 | Overnight, 4ºC | Mouse | MAB10391 |
| Smad3^1^ | 1/100 | Overnight, 4ºC | Rabbit | ab28379 |
| Smad4^1^ | 1/100 | Overnight, 4ºC | Rabbit | ab208804 |
| Smad7^1^ | 1/200 | Overnight, 4ºC | Rabbit | ab216428 |
| THBS1^1^ | 1/200 | Overnight, 4ºC | Mouse | ab85762 |
| TGFβ1^6^ | 1/200 | 60 min, RT | Rabbit | SC-146 |
| TGFβRI^1^ | 1/50 | 60 min, RT | Rabbit | ab31013 |
| TGFβRII^1^ | 1/100 | 30 min, RT | Rabbit | ab28382 |

^1^AbCam, Cambridge, UK; ^2^Novus Biologicals LLC, USA; ^3^Gene Tex, CA, USA; ^4^Abcepta, San Diego, USA; ^5^Abnova, Taipei City, Taiwan, China; ^6^Santa Cruz Biotech., Santa Cruz, CA, USA. RT = room temperature.
